# Extremely Foldable and Highly Transparent Nanofiber-Based Electrodes for Liquid Crystal Smart Devices

**DOI:** 10.1038/s41598-018-29940-3

**Published:** 2018-08-01

**Authors:** In Chul Kim, Tae-Hyung Kim, Seung Hee Lee, Byoung-Suhk Kim

**Affiliations:** 1 Department of Organic Materials & Fiber Engineering, Chonbuk National University, 567 Baekje-daero, Deokjin-gu, Jeonju-si, Jeollabuk-do, 54896 Republic of Korea; 2 Graduate School of Flexible & Printable Electronics Engineering, Chonbuk National University, 567 Baekje-daero, Deokjin-gu, Jeonju-si, Jeollabuk-do, 54896 Republic of Korea; 3 Department of Polymer-Nano Science and Technology, Chonbuk National University, 567 Baekje-daero, Deokjin-gu, Jeonju-si, Jeollabuk-do, 54896 Republic of Korea; 4 Department of BIN Convergence Technology, Chonbuk National University, 567 Baekje-daero, Deokjin-gu, Jeonju-si, Jeollabuk-do, 54896 Republic of Korea

## Abstract

The nylon 6 nanofiber-reinforced cellulose acetate (NF-r-CA) film as a fiber-based transparent substrate is used to develop the highly transparent electrodes with excellent durable and extremely foldable properties. Mechanical properties of the NF-r-CA films are greatly improved, suggesting that the nanofibers provide an effective reinforcement. The NF-r-CA transparent films show smooth surface morphologies (R_RMS_ ~ 27 nm) than as-spun nylon 6 nanofiber membrane, indicating the successful infiltration of cellulose acetate into the voids of nylon nanofiber membranes. The NF45-r-CA electrodes prepared using AgNWs concentration of 0.025 wt% and electrospinning time of 45 min are highly transparent (~90%), lower sheet resistance (~24 Ω sq^−1^) and mechanically robust (59.7 MPa). The sheet resistance of NF45-r-CA electrodes remains almost constant, and the change ratio is less than 0.01% even after a repeated bending test of 10,000 cycles (bending radius ~1 mm), whereas ITO electrode shows gradual increase in sheet resistance and then eventually no electrical signal at about 270 cycles. We also demonstrate the successful fabrication of the foldable polymer-disperse liquid crystal film utilizing highly transparent NF45-r-CA electrode, which shows outstanding working stability after bending test of 500 cycles at an extreme bending radius of 1.5 mm.

## Introduction

In recent years, flexible transparent electrodes have attracted great attention in many wearable optoelectronic devices, such as touch screens^1,2^, organic light-emitting diodes (OLEDs)^3,4^, solar cells^5,6^, electronic skins^7^, etc. The basic requirements for foldable transparent electrodes are optical transparency, low electrical resistance, and high level of extreme bending toughness without significant decrease in the electrical performance. Generally, resistivity and optical transmittance follow opposite trends^8^. It is therefore important to achieve the optimized balance between the electrical resistivity and optical transmittance in order to get a highly conductive transparent electrode. Traditionally, commercial indium-tin oxide (ITO) electrodes have been widely used in transparent conducting optoelectronic devices^9–11^. However, there are still some disadvantages of ITO electrodes in flexible electronic applications, such as the scarcity of indium, the high cost of manufacturing processing, and their mechanical brittleness, which prompt the research for the alternative materials to replace the ITO electrodes for next generation optoelectronic flexible devices^12^. So far, several promising materials for flexible transparent electrodes, such as conducting polymers^13–15^, carbon nanotube (CNT)^16,17^, graphene^18–20^, metal nanowire^20,21^, metal nanotrough network^22^, and their hybrid materials^23–25^ have been used to fabricate highly transparent and flexible electrodes with low resistance and high flexibility. However, most of the transparent films used as a flexible substrate are polymer-based film, such as polyethylene terephthalate (PET), polyethylene naphthalate (PEN), which are not extremely foldable and therefore are limited for the ultimate folding condition, for instance, even 1 mm in bending radius. In particular, silver nanowires (AgNWs) are one of the most promising transparent conductive electrode materials because they offer a low resistance, good optical transparency, and high mechanical flexibility^26,27^. AgNWs dispersed in a solvent can be deposited onto larger-area flexible substrates using simple solution coating techniques, such as spin coating, spray coating, or Meyer-rod coating^28,29^.

On the other hand, liquid crystal displays (LCDs) are widely used in display market, however, they have intrinsic difficulties in realizing flexible LCDs because liquid crystals are fluid materials and maintaining a liquid crystal layer between two brittle ITO electrodes is difficult under external pressure, bending distortion, and mechanical shock^30–33^. Nevertheless, the mixtures of polymer and liquid crystals, such as the polymer dispersed liquid crystals (PDLCs) and optically isotropic liquid crystals (OILCs), can overcome these drawbacks to apply flexible LC devices because of embedded LC droplets in polymer matrix^34,35^. Recently, the PDLCs have been used in switchable electronic device applications such as the displays^36^, smart window^37^, micro-lenses^38^, data storage^39^, and sensor^40^, however, it still has limitation in applications because conventional PDLCs use transparent ITO and thus its flexibility is limited.

Herein, we report a novel approach for the fabrication of nanofiber-reinforced highly transparent and extremely foldable electrodes. The application of nanofibers as reinforcement in the composite is highly effective in the preparation of optically transparent fiber-reinforced plastics (FRPs)^41^. Thus, these FRPs are preferable base materials for the fabrication of highly transparent and robust electrodes. Firstly, nanofiber-reinforced cellulose acetate (NF-r-CA) thin films were prepared using a simple solution casting technique. Then, Ag nanowires were spin-coated on the obtained transparent fibrous films to produce Ag nanowire networks by varying spin-coating conditions, such as the concentration of Ag nanowire dispersion, spinning rate and time, etc. After the deposition of Ag nanowire networks on the transparent fibrous films, highly transparent and extremely foldable nanofiber-reinforced electrodes were obtained, which showed the optical transmittance of ~ 90% and the sheet resistance (Rs) of 20~30 Ω sq^−1^ even under extreme bending radius of 1 mm for 10,000 cycles. As far as we know, there is no report for the fabrication and its electric-optical properties of the extremely foldable PDLC film prepared using the fiber-based electrodes. In this work, we report the fabrication of the flexible PDLC devices using nanofiber-reinforced highly transparent and extremely foldable electrodes and its working performances before and after bending tests at an extreme bending radius of 1.5 mm.

## Experimental

### Materials

AgNWs (0.1 wt% dispersed in water, 40 nm in diameter, 20 μm in length) were purchased from Nanotech & Beyond, Daejeon, Republic of Korea. Nylon 6 (PA-6, M_w_ = 104.83 kDa) and cellulose acetate (CA, M_n_ = 50 kDa, acetyl 39.7 wt%) were purchased from Sigma Aldrich. Polyethyleneimine (PEI), formic acid, isopropyl alcohol (IPA) and dimethylformamide (DMF) were purchased from Junsei Co. Ltd. (Japan). The nematic liquid crystal E7 (∆n = 0.2249, n_e_ = 1.7512 at 25 °C, n_o_ = 1.5268 at 25 °C, Δε = 13.8, T_NI_ = 59 °C) was purchased from Merck Advanced Technology, Republic of Korea. UV-curable monomer Norland Optical Adhesive (NOA65, n_e_ = 1.524) were purchased from Norland Products Inc., USA. All the reagents were of analytical grade and used without further purification. All the aqueous solutions were prepared with deionized water (18.2 MΩ·cm, Elga DI water system).

### Preparation of highly transparent NF-r-CA films

The solution of nylon 6 was prepared using formic acid at the concentration of ~6.0 wt%. Then, the PA-6 solution was electrospun at 9~10 kV (NanoNC power supply, Republic of Korea) at a tip-to-collector distance of 15 cm at 20 °C and at relative humidity of 40~50%. The electrospinning was performed for 15~60 min and the PA-6 electrospun nanofibers were collected on ITO glass (iTASCO, Seoul, Republic of Korea) and vacuum-dried at room temperature for 24 h. Then, the obtained PA-6 nanofiber membrane was coated with cellulose acetate solution dissolved in DMF at the concentration of 10.0 wt%, and kept for drying at room temperature for 1 h followed by drying at room temperature under vacuum for 24 h. Afterwards, the nanofiber-reinforced transparent films were peeled off from the ITO glass and stored at room temperature until further use. Here, we expect that the PA-6 nanofiber reinforced CA (NF-r-CA) film becomes optically transparent, due to the similar RI values (PA-6: 1.530, CA: 1.475)^42^ of the PA-6 nanofibers (*as a reinforcement*, PA-6) and the cellulose acetate (*as a matrix*, CA). In order to investigate the effects of the optical transmittance on the nanofiber contents in PA-6 nanofiber membrane, we have also prepared the NF-r-CA films with different spinning times for 15, 30, 45 and 60 min, which were denoted as NF15-r-CA, NF30-r-CA, NF45-r-CA and NF60-r-CA, respectively.

### Preparation of AgNWs-coated highly transparent and extremely foldable electrodes

The obtained PA-6 nanofiber-reinforced transparent film was cut into the pieces having the dimension of 2.5 cm × 4 cm. A solution of AgNWs dispersed in water was spin-coated onto the transparent NF-r-CA substrate and heated in oven at 60 °C overnight to remove any remaining solvents as well as improve the adhesion between the AgNWs and the substrate. Here, we should also note that the adhesive layer of PEI was pre-coated before AgNWs coating (*PEI was often used as an initial adhesive layer for built-up of the multilayer polyelectrolyte thin films via a Layer-by-Layer self-assembly*^43^). The multi-step spin-coating method was developed to prepare uniform and interconnected AgNWs networks on the transparent NF-r-CA substrate. At first, as-received AgNWs dispersion of 0.1 wt% was diluted to 0.025% and 0.05%, respectively, and then used for spin-coating process. Each AgNW dispersion (ca. 5 drops) was spin-coated for 2,500 rpm for 45 sec and 3,000 rpm for 45 sec, respectively.

### Fabrication of foldable PDLC films

The PDLC film was prepared using the highly transparent and foldable electrodes. For comparison, the commercial ITO electrode was used. The PDLC film was composed of 60 wt% nematic liquid crystal, E7 and 40 wt% UV-curable monomer, Norland Optical Adhesive 65 (NOA65). We have fabricated two types of PDLC films using either ITO films or highly transparent and foldable NF-r-CA films as top and bottom electrodes. To fabricate these devices, initially we have placed ITO or NF-r-CA film as a bottom electrode on the glass substrate using thermal release tape. To maintain film thickness, the spacer of 10 µm was dispersed in IPA and sprayed on the surface of each electrode films. Then, the homogeneous mixture of E7 LC and NOA65 was placed on the surface of electrode film. Afterwards, the bottom electrodes were assembled with the same kind of top electrode (ITO or NF-r-CA) film to fabricate the PDLC film. The phase separation was obtained by exposing the films to the 365 nm UV radiation (HAMAMATSU, LC8 L9588, Japan) with the intensity of 10 mW cm^−2^ for 450 s at room temperature. Finally, the fabricated PDLC film was peeled off from the thermal release tapes at 100 °C. The detailed schematic illustration for the fabrication of the PDLC film using ITO and NF45-r-CA electrodes is shown in Supplementary Fig. S1.

### Characterization

A field-emission scanning electron microscope (FE-SEM, HITACHI SU-70) was used to observe the morphologies of the prepared samples. The optical transmittance was obtained by using a UV-visible spectroscopy (Shimadzu UV-1800, Tokyo, Japan). The bending test was performed using a Radius Bending Tester-JIRBT-610 interfaced with a Keithley-6221 DC/AC current source. In the bending test, the edges of the transparent NF-r-CA electrodes were fixed between two clamps horizontally. And then the electrode was bent by pushing the two clamps together up to a bending radius of 1 mm. Mechanical properties were performed by using a universal testing machine (AG-5000G; Shimadzu Co., Japan) under a cross-head speed of 5 mm min^−1^ at room temperature. In accordance with ASTM D-638, the samples were prepared in the shape of a dumbbell, and then tensile tests were conducted on at least 3~5 specimens and the average values were reported. The flexibility performance of both PDLC films was evaluated by characterizing the electric-optical properties before and after bending tests of both PDLC films with the curvature of 1.5 mm. The voltage-normalized transmittance (V-T curve) characteristics of the fabricated PDLC films were measured using a LC measurement system (LCMS-200, SESIM Photonic Tech., Republic of Korea). Polarized optical microscope (POM) (Nikon, ECLIPSE E600W POL, Japan) was used to observe the droplets of each fabricated PDLC films at V_off_ and V_on_ states.

## Results and Discussion

**Figure 1:**
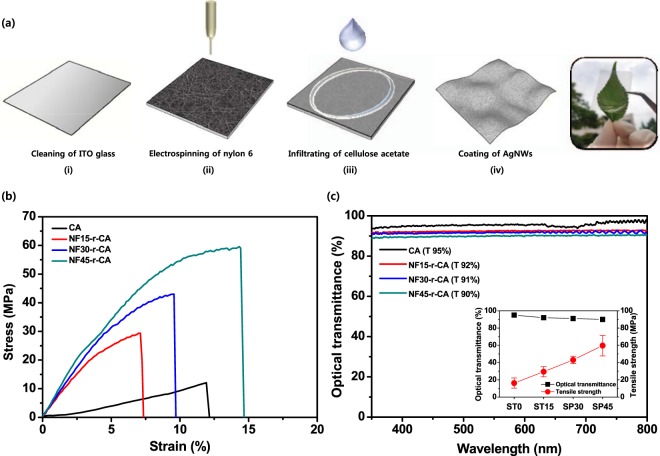
Schematic illustration of the fabrication procedure for the highly transparent fiber-based electrodes. (**a**) Fabrication process steps for the highly transparent fiber-based electrodes with excellent durable and foldable properties. Stress-strain curves (**b**) and optical transmittance (**c**) of bare CA film and NF-r-CA films (NF15-r-CA, NF30-r-CA and NF45-r-CA films) with different nanofiber contents at room temperature. The right inset shows the relationship between the optical transmittance and the tensile strength of the NF-r-CA films with different nanofiber contents.

**Figure 2:**
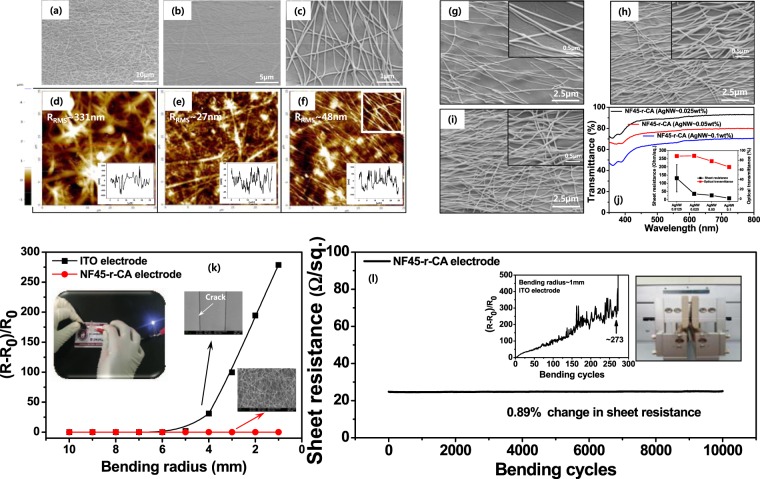
FE-SEM (**a–c**) and AFM (**d–f**) images of as-spun nylon 6 nanofiber membrane, the NF45-r-CA film, and AgNWs-coated transparent NF45-r-CA electrode. The inset in AFM images shows the profiles marked on the image, corresponding to the red line in the image. (**g–i**) Surface morphologies (g: 0.025%, h: 0.05%, i: 0.1%) and optical transmittance (**j**) of the NF45-r-CA transparent electrodes with different concentrations of AgNWs. The right inset shows the relationship between the sheet resistance and the optical transmittance of the NF45-r-CA transparent electrodes with different concentrations of AgNWs. (**k**) Relative change in the sheet resistance of the ITO and NF45-r-CA electrodes as a function of bending radius. The left inset shows visual inspection of the NF45-r-CA electrodes after folding, suggesting an excellent foldable property, and SEM images of each ITO and NF45-r-CA electrodes after bending test. (**l**) Repeated bending test of the NF45-r-CA electrodes for 10,000 cycles at an extreme bending radius of 1 mm. The right inset shows the relative change in the sheet resistance of the ITO electrode during the repeated bending test and visual inspection of bending test.

Figure 1a schematically illustrates the fabrication procedure for the fiber-based highly transparent and foldable electrodes. Firstly, nylon 6 nanofibers were deposited on the cleaned ITO glass (i) by solution electrospinning (ii), and then cellulose acetate solution was poured into nylon 6 nanofiber membrane to produce the highly transparent NF-r-CA film (optical transmittance at 550 nm: 90~92%) using a simple solution casting technique (ii). Afterwards, AgNWs were spin-coated on the transparent NF-r-CA films, which resulted in highly conductive and transparent electrodes (iv). In addition, it can be expected that the nanofiber-reinforced NF-r-CA film used as a transparent substrate exhibits excellent flexibility, durability and elasticity towards ultimate bending, compared to bare cellulose acetate films. Figure 1b shows the stress-strain curves of bare CA film and NF-r-CA films with different nanofiber contents at room temperature. Bare CA film exhibited a peculiar characteristic of thermoplastic elastomers, in that the stress-strain behaviors show linear elasticity as their intrinsic material properties, as shown in Fig. 2b. In contrast, the NF-r-CA films showed dramatically enhanced mechanical properties. The higher nanofiber content is, the better mechanical properties are. For instance, the tensile strength of the NF-r-CA films were gradually increased to 29.4, 43.0 and 59.7 MPa as increasing the nanofiber contents (that is, by increasing the electrospinning time from 15, 30 and 45 min), respectively. The NF-r-CA films prepared from the nylon nanofibers electrospun for 60 min (NF60-r-CA film) showed further improvement in mechanical properties, while optical transmittance was largely decreased (less than 88%). This is limited its applications in the highly transparent electronics. Moreover, the toughness, defined as the energy absorbed by the films until breaking, for the NF45-r-CA film showed about 9 times higher value (~586.0 kN/mm) than that of the bare CA film (~65.7 kN/mm). As a result, the strength and toughness of the NF-r-CA films were greatly improved, suggesting that the nanofibers provide an effective reinforcement. As a result, we have chosen the NF-r-CA films prepared from the nylon nanofibers electrospun for 45 min (NF45-r-CA film) as the optimized transparent substrate for the development of the highly conductive and mechanically durable electrodes. The detailed Young’s modulus, tensile strength and toughness of the NF-r-CA films with different nanofiber contents were summarized in Table 1. Figure 1c shows the optical transmittance of bare CA film and NF-r-CA films with different nanofiber contents. The optical transmittance decreased a little as increasing the nanofiber contents and then kept more than 90% while the tensile strength increased linearly (inset in Fig. 1c). Importantly, it should be noted that all nanofiber-reinforced NF-r-CA films were highly transparent (>90%).

**Table 1:** The detailed Young’s modulus, tensile strength and toughness of the bare CA and NF-r-CA films (NF15-r-CA, NF30-r-CA and NF45-r-CA films) with different nanofiber contents.

	Young’s modulus (MPa)	Tensile strength (MPa)	Toughness (kN/mm)
Cellulose (CA)	192.7	16.2	65.7
NF-r-CA 15	756.9	29.4	134.1
NF-r-CA 30	794.4	43.0	262.5
NF-r-CA 45	914.9	59.7	586.0

Figure 2a–f shows a comparison (FE-SEM and AFM) of the surface morphologies of as-spun nylon 6 nanofiber membrane, the NF-r-CA film, and AgNWs-coated transparent NF-r-CA electrode. As seen in Fig. 2a, the fabricated nylon 6 nanofibers exhibited randomly oriented morphologies, with an average fiber diameter of 120 ± 25 nm. On the other hand, the NF-r-CA film showed smooth surface morphologies (Fig. 2b) than as-spun nylon 6 nanofiber membrane, indicating the successful infiltration of the cellulose acetate into the voids of nylon nanofiber membranes with preserving its fibrous structure. Figure 2c shows the surface morphologies of the NF-r-CA transparent electrodes coated with AgNWs. As described in Fig. 2c, it can be clearly seen that the AgNWs were well coated to the surface of the NF-r-CA film. An interesting point is that the roughness of the NF-r-CA transparent film was dramatically decreased by filling such voids in the nanofiber membranes as well as flattening the rough surface, which will be beneficial for the fabrication of liquid crystal device. This surface roughness was also confirmed by the AFM measurements. As shown in Fig. 2d,e, as-spun nylon 6 nanofiber membrane showed rough surface roughness (R_RMS_ ~ 331 nm), while the NF-r-CA film showed much smoother roughness (R_RMS_ ~ 27 nm), indicating the successful infiltration of the cellulose acetate into the voids of nylon nanofiber membranes, which was well coincided with SEM results. After the AgNWs were coated on the surface of the NF-r-CA film, surface roughness was not largely varied, as seen in Fig. 2f.

In general, as-spun nanofiber membranes are opaque or non-transparent due to light scattering, while it can become transparent by filling such voids in the nanofiber membranes (refractive index (RI) of nylon 6~1.530)^42^ with the polymer (RI of cellulose acetate ~1.475)^42^ having the similar RI value, which resulted in the highly transparent NF-r-CA films with an optical transmittance of 90~92% at 550 nm, depending on the nanofiber contents. Indeed, optical transmittance of as-spun nylon 6 nanofiber mats showed drastically lower transmittance and, as expected, dependent on the spinning time ranging from 15 to 60 min, mostly due to the light scattering (Supplementary Fig. S2a). Here, the spinning conditions with different spinning times of 15, 30, 45 and 60 min were denoted as ST15, ST30, ST45 and ST60, respectively. On the other hand, interestingly, optical transmittance of the NF-r-CA films was dramatically enhanced, mainly due to the filling of the voids in the nanofiber membranes with the CA polymer (Supplementary Fig. S2b). In order to study the effects of RI value on the optical transmittance in the nanofiber-reinforced transparent films, various polymers (PVDF: polyvinylidene fluoride, PVAc: polyvinyl acetate, CA, PAA: polyacrylic acid, PS: polystyrene) with different RI values were used. As seen in Fig. S3a, the similar RI value produced the highly transparent films, while the mismatch of RI value gave poor optical transmittance (Supplementary Fig. S3b).

To further investigate the effects of the AgNWs concentration on the optical transmittance and sheet resistance of the NF-r-CA film electrodes, we have studied the relationship between the optical transmittance and sheet resistance of the fiber-based transparent electrodes prepared using various AgNWs concentrations. The concentration of AgNWs, ranging from 0.025 to 0.1%, was further controlled in order to optimize the relationship between average sheet resistance and optical transmittance because optical transmittance and sheet resistance was inversely proportional to each other. Figure 2g–i shows the surface morphologies of the NF-r-CA transparent electrodes with different concentrations of AgNWs. As shown in tilted-view FE-SEM images (inset in Fig. 2g–i), it was observed that the AgNWs were impregnably adhered to the NF-r-CA film and therefore it would be difficult to remove it from the underlying NF-r-CA film by any physical means, such as bending, twisting, etc. Here, it should be noted that the pre-coated PEI nanocoating as adhesive layer contributes to the enhanced adhesion between the AgNWs and the NF-r-CA film. Figure 2j shows the optical transmittance of the NF-r-CA transparent electrodes with different concentrations of AgNWs. As increasing the concentration of AgNWs, the sheet resistance and optical transmittance decreased gradually (inset in Fig. 2j). For instance, the sheet resistance and the optical transmittance drastically decreased to ~9.8 Ω sq^−1^ and ~66%, respectively, at the AgNWs concentration of 0.1 wt%. It is also obvious that the presence of higher concentrations of AgNWs on the NF-r-CA transparent films form a conductive network well to decrease the sheet resistivity, while it will block the transmittance of light. Accordingly, the NF-r-CA electrode prepared using AgNWs concentration of 0.025 wt% and electrospinning time of 45 min was highly transparent, lower sheet resistance and mechanically robust.

In addition to the excellent electrical-optical performance, mechanical flexibility of the NF-r-CA transparent electrodes is crucial for the practical applications of flexible optoelectronics. The NF-r-CA transparent electrode was subjected to a repeated bending test. Figure 2k shows the relative change in the sheet resistivity of the NF-r-CA electrodes as a function of bending radius. The relative change in the sheet resistivity is expressed as (R-R_o_)/R_o_; R and R_o_ are the resistivity value before and after bending test. The NF-r-CA electrodes showed fairly good mechanical flexibility even after bending radius of 1 mm. In contrast, the commercial ITO electrode showed a sharp increase of the sheet resistance after bending radius of 5 mm, due to the crack formation, as confirmed by SEM analysis (inset in Fig. 2k). On the other hand, the NF45-r-CA electrode maintained well the AgNW network morphology. It is also significant that the sheet resistivity of the NF-r-CA electrodes shows almost constant, the relative change in the sheet resistivity do not change over 0.1% even after a repeated bending test of 10,000 cycles (Fig. 2l), while the ITO electrode shows gradual increase in the sheet resistance and then eventually no electrical signal after bending test of ca. 270 cycles (inset in Fig. 2l). In addition, considering that AgNWs are coated on one side of the transparent NF-r-CA films, the bending test was also performed in two different ways, such as inner and outer bending, which will experience different mechanical strain bending modes (compressive and tensile strain), respectively. As a result, the NF-r-CA electrodes were not affected by different mechanical strain bending (2~3% change in sheet resistance ratio of after to before repeated bending cycles), implying outstanding bending property and reliability as well as flexibility after repeated bending test (Supplementary Fig. S4).

**Figure 3:**
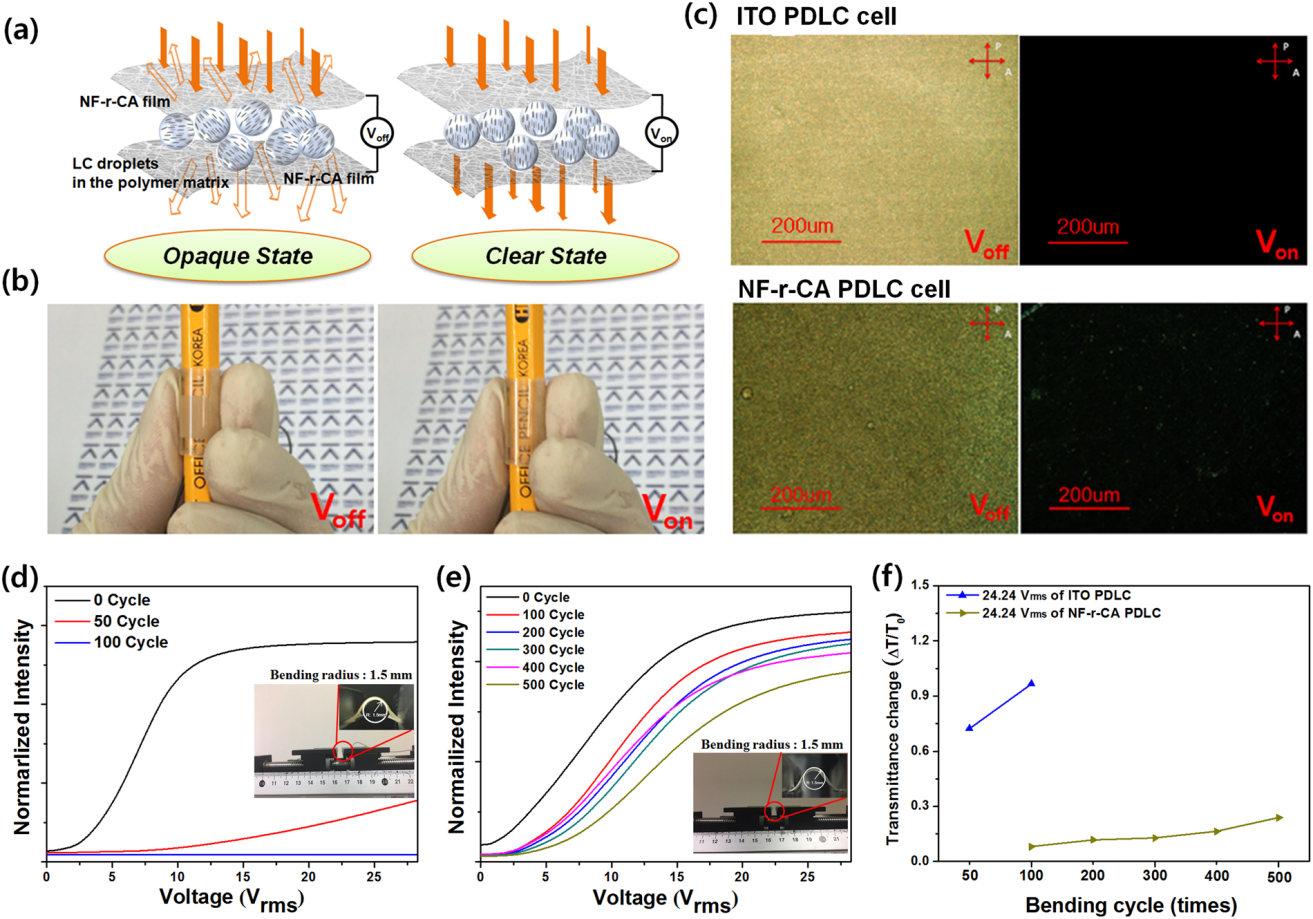
Comparison of PDLC films based on ITO and NF45-r-CA electrodes. (**a**) Schematic of opaque and transparent states of the NF45-r-CA based PDLC film. (**b**) Photographs of the NF45-r-CA PDLC film rolled up pencil at V_off_ (applied voltage ~ 0 V_rms_) and V_on_ (applied voltage ~ 24.24 V_rms_). (**c**) Comparison of POM images for the ITO and NF-r-CA PDLC based PDLC films at V_off_ and V_on_, respectively. (**d–e**) V-T curves for foldable PDLC films prepared using both ITO (**d**) and NF45-r-CA electrodes (**e**) before and after repeated bending tests at an extreme bending radius of 1.5 mm. The inset shows the visual inspection during bending test. (**f**) Relative transmittance change of the ITO and NF-r-CA PDLC based PDLC films at the applied voltages (24.24 V_rms_) after repeated bending tests (50, 100, 200, 300, 400, and 500 cycles).

We have fabricated the PDLC films using the highly transparent and foldable NF-r-CA electrodes via widely adopted polymerization-induced phase separation method. UV curing of the homogeneous mixture of liquid crystal and monomer resulted in the phase separation and the formation of liquid crystal droplets in a polymer matrix. The flexibility and the variation in optoelectric properties of the fabricated PDLC films were investigated under bending tests. The bending tests were performed by bending as-prepared PDLC films to the bending radius of 1.5 mm. Figure 3a shows the schematic representation of working principle and the structure of foldable PDLC films. When there is no external applied voltage, the randomly oriented LC inside the polymer matrix scatters the incident light and causes the PDLC films to appear opaque. However, under the application of vertical electric field, the LC molecules inside the droplets were aligned towards the field direction such that the refractive indices between polymer and ordinary axis of LCs match, resulting in the transparent state, demonstrating the successful display of precise opaque and transparent states at voltage-off (V_off_) and voltage-on (V_on_), respectively. Figure 3b shows the photographs of as-prepared PDLC films rolled up around a pencil. This result also confirms that highly transparent and foldable NF-r-CA electrodes in the PDLC film works well even under bending. Figure 3c shows the POM images of ITO and NF-r-CA-based PDLC films at V_off_ and V_on_. In particular, although there is a light leakage in NF-r-CA PDLC film due to the light scattering from AgNWs, it shows trivial effect on the film working and the film appeared to more or less equally dark level to that of ITO PDLC film at V_on_ (Fig. 3b).

Figure 3d,e show the V-T curves for ITO-based PDLC film and NF-r-CA-based PDLC film with different bending cycles at an extreme bending radius of 1.5 mm. Despite of its comparatively higher driving voltage than the ITO PDLC film, the NF-r-CA PDLC film showed higher electro-optic stability under bending test. Importantly, the NF-r-CA PDLC film could be operated and maintained consistent vertical electric field even after bending test of 500 cycles, whereas ITO PDLC film was stopped working after bending test of merely 50 cycles. As a result, it demonstrated that the NF-r-CA based PDLC film could retain the working stability even after bending test of 500 cycles at an extreme bending radius of 1.5 mm, due to the excellent flexibility of the extreme foldable and highly transparent NF-r-CA electrode compared to ITO electrode.

Figure 3f displayed the transmittance change of the NF-r-CA PDLC-based film with different bending cycles at various applied voltages. The transmittance change can be explained as ΔT/T_o_, where ΔT is the change in the transmittance value after bending test and T_o_ indicates the initial value of transmittance. Under the applied voltage of 24.24V_rms_, ITO PDLC films showed the swift increment of transmittance change in-between 50 and 100 cycles bending tests, whereas gradual trivial increment is noticed in the case of NF-r-CA PDLC films after each 100 cycles bending test till 500 cycles. The significant transmittance change for ITO PDLC film can be attributed to the absence of vertical electric field due to the ITO electrode fracture during the bending test. On the other hand, in the NF-r-CA PDLC film, the orientation of LC in the droplets is changed vertically along the applied electric field direction at 24.24 V_rms_ even after each bending test (100, 200, 300, 400 and 500 cycles) due to the mechanical stability of NF-r-CA electrodes. This result demonstrates the excellent foldable stability of NF-r-CA PDLC films at an extreme bending radius of 1.5 mm.

## Conclusions

The fiber-based highly transparent electrodes with excellent durable and foldable properties were prepared using the nylon 6 nanofiber-reinforced cellulose acetate (NF-r-CA) films. Compared to bare CA film, the obtained NF-r-CA films showed higher mechanical properties, suggesting that the nanofibers provide an effective reinforcement, and smooth surface morphologies (R_RMS_ ~ 27 nm by AFM analysis), indicating the successful infiltration of the cellulose acetate into the voids of nylon nanofiber membranes. The NF-r-CA electrodes prepared using AgNWs concentration of 0.025 wt% and electrospinning time of 45 min was highly transparent (~90% at 550 nm), lower sheet resistance (~24 Ω sq^−1^) and mechanically robust (59.7 MPa in tensile strength). Importantly, the sheet resistance of the NF45-r-CA electrodes showed almost constant sheet resistance value even after a repeated bending test of 10000 cycles at an extreme bending radius of 1 mm. We have also successfully fabricated the foldable PDLC cell using highly transparent NF45-r-CA electrode and demonstrated its excellent working stability after bending test of 500 cycles at an extreme bending radius of 1.5 mm.

## Electronic supplementary material

Supplementary Information

Video-1

Video-2

## References

[1] Bae SK (2010). Roll-to-roll production of 30-inch graphene films for transparent electrodes. Nat. Nanotechnol..

[2] Cho SS (2017). Large-area cross-aligned silver nanowire electrodes for flexible, transparent, and force-sensitive mechanochromic touch screens. ACS Nano.

[3] Meng H (2013). Top-emission organic light-emitting diode with a novel copper/graphene composite anode. Adv. Funct. Mater..

[4] Li N (2013). Efficient and bright organic light-emitting diodes on single-layer graphene electrodes. Nat. Commun..

[5] Kaltenbrunner M (2012). Ultrathin and lightweight organic solar cells with high flexibility. Nat. Commun..

[6] Im HG (2016). Hybrid crystalline-ITO/metal nanowire mesh transparent electrodes and their application for highly flexible perovskite solar cells. NPG Asia Mater..

[7] Núnez CG, Navaraj WT, Polat EO, Dahiya R (2017). Energy-autonomous, flexible, and transparent tactile skin. Adv. Funct. Mater..

[8] Devarayan K, Lei D, Kim HY, Kim BS (2015). Flexible transparent electrode based on PANi nanowire/nylon nanofiber reinforced cellulose acetate thin film as supercapacitor. Chem. Eng. J..

[9] Rider DA (2011). Indium thin oxide nanopillar electrodes in polymer/fullerene solar cells. Nanotechnology.

[10] Tan ZA (2012). High-performance inverted polymer solar cells with solution-processed titanium chelate as electron-collecting layer on ITO electrode. Adv. Mater..

[11] Lu H (2016). A self-powered and stable all-perovskite photodetector-solar cell nanosystem. Adv. Funct. Mater..

[12] Cairns DR (2000). Strain-dependent electrical resistance of tin-doped indium oxide on polymer substrates. Appl. Phys. Lett..

[13] Tai Q (2011). *In situ* prepared transparent polyaniline electrode and its application in bifacial dye-sensitized solar cells. ACS Nano.

[14] Bu C, Tai Q, Liu Y, Guo S, Zhao X (2013). A transparent and stable polypyrrole counter electrode for dye-sensitized solar cell. J. Power Sources.

[15] Ge J, Cheng G, Chen L (2011). Transparent and flexible electrodes and supercapacitors using polyaniline/single-walled carbon nanotube composite thin films. Nanoscale.

[16] Reynaud O (2014). Aerosol feeding of catalyst precursor for CNT synthesis and highly conductive and transparent film fabrication. Chem. Eng. J..

[17] Zhang D (2006). Transparent, Conductive, and flexible carbon nanotube films and their application in organic light-emitting diodes. Nano Lett..

[18] Cai W, Zhu Y, Li X, Piner RD, Ruoff RS (2009). Large area few-layer graphene/graphite films as transparent thin conducting electrodes. Appl. Phys. Lett..

[19] Kim KS (2009). Large-scale pattern growth of graphene films for stretchable transparent electrodes. Nature.

[20] Hecht DS, Hu L, Irvin G (2011). Emerging transparent electrodes based on thin films of carbon nanotubes, graphene, and metallic nanostructures. Adv. Mater..

[21] Guo H (2013). Copper nanowires as fully transparent conductive electrodes. Sci. Rept..

[22] Wu H (2013). A transparent electrode based on a metal nanotrough network. Nat. Nanotechnol..

[23] Kholmanov IN (2012). Improved electrical conductivity of graphene films integrated with metal nanowires. Nano Lett..

[24] Chen R (2013). Co-percolating graphene-wrapped silver nanowire network for high performance, highly stable, transparent conducting electrodes. Adv. Funct. Mater..

[25] Mao L (2014). Flexible silver grid/PEDOT:PSS hybrid electrodes for large area inverted polymer solar cells. Nano Energy.

[26] Li L (2014). A solution processed flexible nanocomposite electrode with efficient light extraction for organic light emitting diodes. Sci. Rep..

[27] Kang H (2016). Flexible and mechanically robust organic light-emitting diodes based on photopatternable silver nanowire electrodes. J. Phys. Chem. C.

[28] Scardaci V, Coull R, Lyons PE, Rickard D, Coleman JN (2011). Spray deposition of highly transparent, low-resistance networks of silver nanowires over large areas. Small.

[29] Hu L, Kim HS, Lee JY, Peumans P, Cui Y (2010). Scalable coating and properties of transparent, flexible, silver nanowire electrodes. ACS Nano.

[30] Li WY (2015). The First Flexible Liquid Crystal Display Applied for Wearable Smart Device. SID Digest.

[31] Sato H, Fujikake H, Iino Y, Kawakita M, Kikuchi H (2002). Flexible Grayscale Ferroelectric Liquid crystal Device Containing Polymer Walls and Networks. Jpn. J. Appl. Phys..

[32] Sato H (2001). Fluorinated Polymer Alignment Layers Formed at Low Temperature for Plastic-Substrate-Based Liquid Crystal Devices. Jpn. J. Appl. Phys..

[33] Sikharulidze D (2005). Nanoparticles: An approach to controlling an electro-optical behavior of nematic liquid crystals. Appl. Phys. Lett..

[34] Malik P, Raina KK (2004). Droplet orientation and optical properties of polymer dispersed liquid crystal composite films. Optical Materials.

[35] *Liquid Crystal Dispersions* (ed. Pual S. Drzaic) Ch. 4, 183–352 (World Scientific, Singapore, 1995).

[36] Sheraw CD, Zhou L, Huang JR, Gundlach DJ, Jackson TN (2002). Organic thin-film transistor-driven polymer-dispersed liquid crystal displays on flexible polymeric substrates. Appl. Phys. Lett..

[37] Khaligh HH, Liew K, Han Y, Abukhdeir NM, Goldthorpe I (2015). A Silver nanowire transparent electrodes for liquid crystal-based smart window. Sol. Energ. Mat. Sol. Cells..

[38] Xiong GR (2009). Phototunable Microlens Array based on Polymer Dispersed Liquid crystals. Adv. Funct. Mater..

[39] Liu YJ, Sun XW (2007). Electrically switchable computer-generated hologram recorded in polymer-dispersed liquid crystal. Appl. Phys. Lett..

[40] Lai YT, Kuo JC, Yang YJ (2014). A novel gas sensor using polymer-dispersed liquid crystal doped with carbon nanotubes. Sens. Actuator A-Phys..

[41] Shams MI, Yano H (2015). Doubly curved nanofiber-reinforced optically transparent composites. Sci. Rep..

[42] Scientific Polymer Products (ISO 9001: 2008REGISTERED), Inc., Refractive Index of Polymers by Index. http://scientificpolymer.com/technical-library/refractive-index-of-polymers-by-index (2015).

[43] Vinogradova OI, Lebedeva OV, Kim BS (2006). Mechanical behavior and characterization of microcapsules. Annu. Rev. Mater. Res..

