# Supplementary material for: Extremely Foldable and Highly Transparent Nanofiber-Based Electrodes for Liquid Crystal Smart Devices
Source: Sci Rep. 2018 Aug 1;8:11517. doi: 10.1038/s41598-018-29940-3 (PMC6070569; doi:10.1038/s41598-018-29940-3)
Supplement: Supplementary file 1 — Supplementary Information [file 41598_2018_29940_MOESM1_ESM.docx]

**Supporting Information**

Extremely Foldable and Highly Transparent Nanofiber-Based Electrodes for Liquid Crystal Smart Devices

**In Chul Kim^1†^, Tae-Hyung Kim^2†^, Seung Hee Lee^3,4^ & Byoung-Suhk Kim^1,4^**

^1^Department of Organic Materials & Fiber Engineering, Chonbuk National University, 567 Baekje-daero, Deokjin-gu, Jeonju-si, Jeollabuk-do 54896, Republic of Korea

^2^Graduate School of Flexible & Printable Electronics Engineering, Chonbuk National University, 567 Baekje-daero, Deokjin-gu, Jeonju-si, Jeollabuk-do 54896, Republic of Korea

^3^Department of Polymer-Nano Science and Technology, Chonbuk National University, 567 Baekje-daero, Deokjin-gu, Jeonju-si, Jeollabuk-do 54896, Republic of Korea

^4^Department of BIN Convergence Technology, Chonbuk National University, 567 Baekje-daero, Deokjin-gu, Jeonju-si, Jeollabuk-do 54896, Republic of Korea

*Corresponding Authors: Seung Hee Lee: [lsh1@jbnu.ac.kr](mailto:lsh1@jbnu.ac.kr), Byoung-Suhk Kim; E-mail: kbsuhk@jbnu.ac.kr

^†^ Authors are equally contributed.

A running title: Foldable and transparent nanofiber-based electrodes


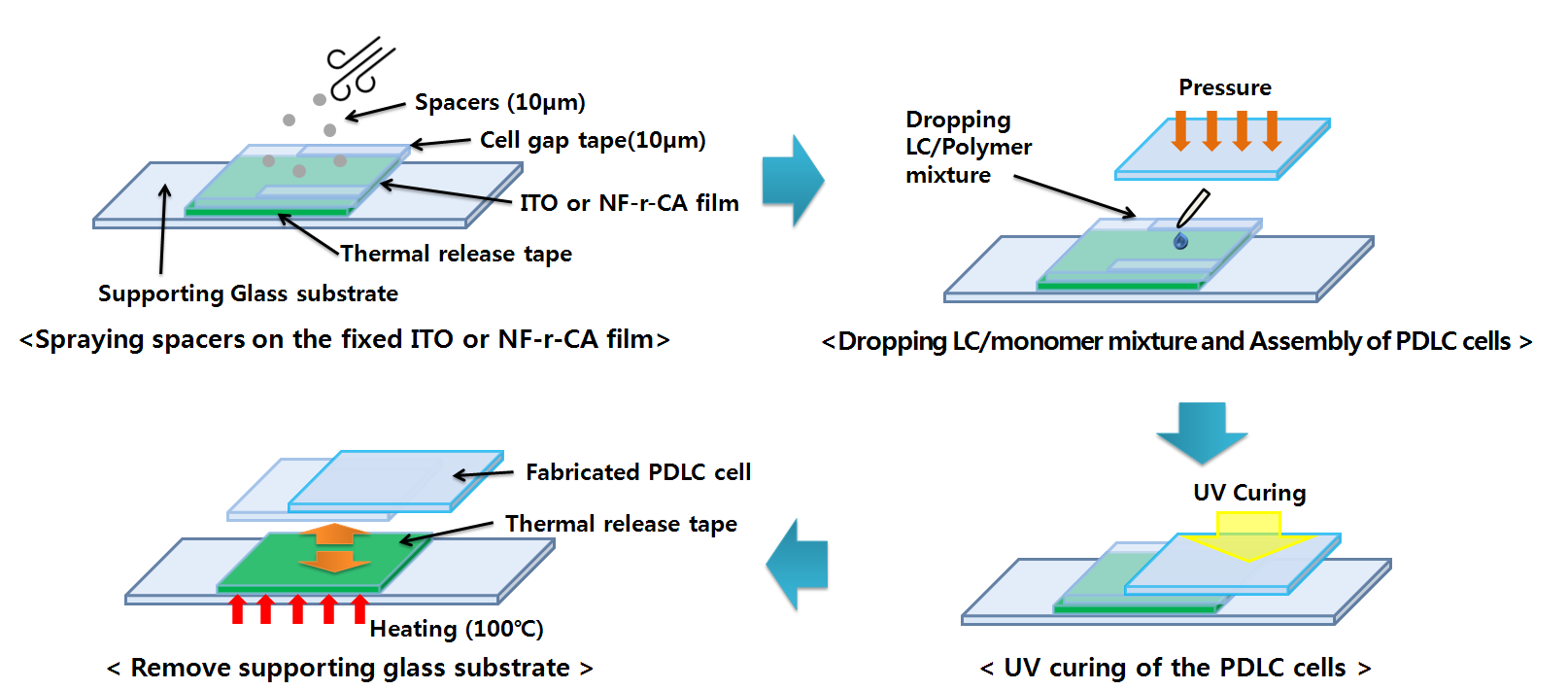


**Figure S1.** Schematic illustration for the fabrication step of the PDLC film using ITO and NF45-r-CA electrodes.

**Figure S2.** The optical transmittance of as-spun nylon 6 nanofiber membranes (a; ST15, ST30, ST45 and ST60) and the corresponding NF-r-CA films (b; NF15-r-CA, NF30-r-CA, NF45-r-CA and NF60-r-CA) with different spinning times, ranging from 15 to 60 min.

**Figure S3.** The optical transmittance of nylon 6 nanofiber-reinforced films prepared using various polymers (PVDF, PVAc, CA, PAA, PS) (a) and its relative ratio of refractive index values to the nylon 6 (b).

**Figure S4.** The sheet resistance change of the NF45-r-CA electrode during different mechanical strain bending (compressive and tensile strain) tests at an extreme bending radius of 1 mm.

**Movie S1.**

**
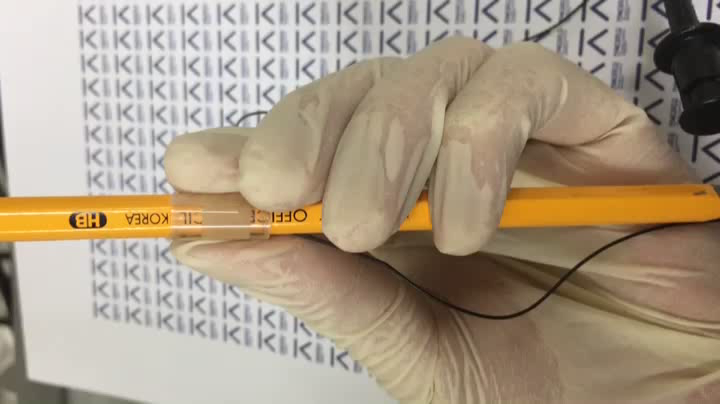
**

**Movie S2.**

**
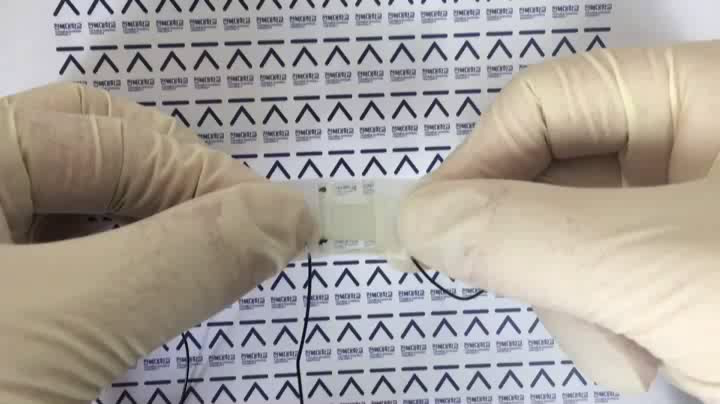
**
